# Adverse Childhood Experiences, Family Functioning, and Mental Health in Vulnerable Women from Santa Marta, Colombia

**DOI:** 10.1192/j.eurpsy.2026.11670

**Published:** 2026-07-31

**Authors:** K. Munera-Luque, Z. Miranda-Sánchez, D. Sánchez-Pabón, Ó. Rodríguez-Vega

**Affiliations:** Magdalena, Universidad Cooperativa de Colombia, Santa Marta, Colombia

## Abstract

**Introduction:**

Adverse childhood experiences significantly impact mental health and family functioning in adulthood. Understanding these interrelations is essential to designing interventions that strengthen family support networks and mitigate associated psychological consequences.

**Objectives:**

To analyze the relationship between adverse childhood experiences, family functioning, and mental health in vulnerable women residing in Santa Marta, Colombia.

**Methods:**

A quantitative, correlational, cross-sectional study was conducted using a convenience sample (n = 170). A sociodemographic questionnaire, the Family APGAR, the Adverse Childhood Experiences (ACE) questionnaire, and the Self-Reporting Questionnaire (SRQ) were applied. Statistical analysis included descriptive statistics and Spearman’s correlations.

**Results:**

The participants’ mean age was 41.2 years (SD = 11.2). The mean scores (SD) obtained were: APGAR 15.69 (4.96), ACE 2.41 (3.72), SRQ-Depression 3.53 (4.62), Distress 3.03 (2.94), Alcohol use 0.59 (2.03), and Suicidal ideation 0.16 (0.45). A total of 58.8% of participants reported a perception of functional family (APGAR ≥ 17), while 7.6% presented a high burden of adverse experiences (ACE ≥ 7). Significant negative correlations were found between APGAR and Depression (Rho = –0.175; p = 0.022), Psychosis (Rho = –0.152; p = 0.048), and Suicidal ideation (Rho = –0.183; p = 0.017). Likewise, ACE scores were positively correlated with Depression (Rho = 0.386; p < 0.001), Distress (Rho = 0.426; p < 0.001), Psychosis (Rho = 0.414; p < 0.001), and Suicidal ideation (Rho = 0.240; p = 0.002).

**Conclusions:**

Adverse childhood experiences are associated with impaired family functioning and increased psychological symptoms in adulthood. These findings highlight the importance of implementing comprehensive psychosocial programs that address these issues both preventively and therapeutically.

**Disclosure of Interest:**

None Declared

